# Outcome of Patients With Cancer‐Associated Pulmonary Embolism: Results From the Regional Pulmonary Embolism Registry

**DOI:** 10.1002/cam4.70886

**Published:** 2025-04-25

**Authors:** Sonja Salinger, Aleksandra Kozic, Boris Dzudovic, Bojana Subotic, Jovan Matijasevic, Marija Benic, Vladimir Miloradovic, Ema Jevtic, Tamara Kovacevic‐Preradovic, Ljiljana Kos, Nebojsa Bulatovic, Bjanka Bozovic, Irena Mitevska, Marijan Bosevski, Ana Kovacevic‐Kuzmanovic, Milos Svircev, Aleksandar Neskovic, Bojan Mitrovic, Srdjan Kafedzic, Slobodan Obradovic

**Affiliations:** ^1^ Clinic of Cardiology Clinical Center Nis Nis Serbia; ^2^ Faculty of Medicine University of Nis Nis Serbia; ^3^ Medical Faculty of the Military Medical Academy University of Defense Belgrade Serbia; ^4^ Clinic of Emergency Internal Medicine Military Medical Academy Belgrade Serbia; ^5^ Clinic of Cardiology Military Medical Academy Belgrade Serbia; ^6^ Department for Intensive Pulmonary Care Institute for Pulmonary Diseases Vojvodina Novi Sad Serbia; ^7^ Faculty of Medicine University in Novi Sad Novi Sad Serbia; ^8^ Clinic of Cardiology Clical Center Kragujevac Kragujevac Serbia; ^9^ Faculty of Medical Sciences University of Kragujevac Kragujevac Serbia; ^10^ Clinic of Cardiology Clinical Center Banja Luka Banja Luka Republic of Srpska, Bosnia and Herzegovina; ^11^ Faculty of Medicine University of Banja Luka Banja Luka Republic of Srpska, Bosnia and Herzegovina; ^12^ Clinic of Cardiology Clinical Center Podgorica Podgorica Montenegro; ^13^ Faculty of Medicine University of Montenegro Podgorica Montenegro; ^14^ Clinic of Cardiology Clinical Center Skopje Skopje North Macedonia; ^15^ Medical Faculty Skopje University Ss Cyril and Methodius Skopje North Macedonia; ^16^ Department for Internal Medicine General Hospital Pancevo Pancevo Serbia; ^17^ Clinic of Cardiology Clinical Center Zemun Belgrade Serbia; ^18^ School of Medicine University of Belgrade Belgrade Serbia

**Keywords:** cancer, outcome, presentation, pulmonary embolism

## Abstract

**Background:**

Newly or already diagnosed cancer might significantly influence the clinical presentation, outcome, and therapy of acute pulmonary embolism (PE).

**Methods:**

Out of 1745 patients with acute PE, 66 patients were diagnosed with cancer during an initial hospitalization due to acute PE (where PE was the first clinical manifestation of cancer), 165 patients had known cancer treated in the last 6 months, and 1514 patients had acute PE without known or suspected cancer. The primary end‐point of the present study was all‐cause hospital death. The secondary end‐points were the proportion of patients treated with thrombolysis and who had severe disease, and the ocurrence of major or clinically relevant nonmajor bleeding.

**Results:**

Patients with PE as the first presentation of cancer had the highest hospital mortality rate compared to the other two groups (HR for the mortality rate in patients without cancer as a reference, adjusted to four‐stratum mortality risk, and Charlson's comorbidity index was 3.440; 95% confidence interval (CI), 1.795–6.591; *p* < 0.001). Patients with known cancer before PE had a significantly lower chance of being treated with thrombolysis than patients without cancer (OR, 0.523; 95% CI, 0.339–0.807; *p* = 0.003); additionally, this difference was attenuated but remained when the OR was adjusted to age (OR, 0.542; 95% CI, 0.351–0.838; *p* = 0.006). Patients with known cancer had a higher frequency of high‐risk PE compared with patients without cancer (18.2% vs. 12.8%; *p* < 0.001). Patients with PE as the first manifestation of cancer had a higher frequency of intermediate‐high‐risk PE than those without (36.4% vs. 30.9%; *p* < 0.001). There was no significant difference in bleeding during hospitalization between groups.

**Conclusion:**

Patients with cancer had a more severe presentation of acute PE than patients without. Furthermore, patients with PE as the first manifestation of cancer had the highest hospital mortality rate, and patients with known cancer were least likely to be treated with thrombolysis.

## Graphical abstract

The results of our investigation have shown that patients with newly diagnosed malignant disease with acute PE and patients with known malignant disease and PE had much higher hospital mortality and more severe acute PE presentation than patients without malignant disease. We have also shown that patients with known malignant disease had a lower chance of thrombolysis treatment than patients without malignant disease.

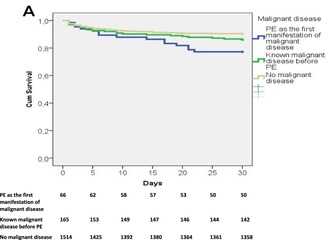

## 1 Introduction

The development of numerous new treatment modalities has led to the association between cancer and venous thromboembolism (VTE) becoming more complex and remains one of the greatest challenges in the management of patients with cancer [1]. Patients with cancer possess a higher risk for the development of VTE, and there has been a recent increase in the incidence of VTE within this vulnerable group of patients [2, 3, 4, 5, 6]. The association between cancer and pulmonary embolism (PE) involves all three factors of Virchow's triad through several pathophysiological mechanisms [2, 3, 4]. However, the frequency of VTE in patients with cancer is not consistent across different studies, as it depends on factors such as the cancer type and stage, duration of follow‐up, treatment exposure, and the detection method used [2, 3, 4, 5, 6].

Many anticancer drugs can provoke thrombosis [7, 8, 9, 10, 11, 12]. Classic chemotherapy, antihormonal therapy, and immune therapy can provoke a hypercoagulable state, damage the endothelium and venous wall, and cause venous thromboembolism [7, 8, 9, 10, 11, 12].

Research indicates that there is an association between cancer aggressiveness and the formation of blood clots, with metastatic cancer being identified as one of the most significant predictors of VTE [2, 4, 5, 13]. It is hypothesized that VTE/PE may be an early indicator of cancer, and individuals who survive this condition can pose a significant challenge for follow‐up diagnostic and treatment procedures [5]. There is a lack of comparative data on the inpatient outcomes of cancer‐associated PE and cancer‐free patients with PE, despite the published studies highlighting the incidence of PE in the general population and the elevated risk of VTE in patients with cancer. The short‐term prognosis and the outcome of patients with VTE/PE as the first manifestation of cancer are of great importance. It could be stratified first as an unprovoked PE but with an unfavorable outcome due to an unknown underlying disease. The clinically important question is whether to look for an occult or more advanced cancer and who may benefit from such investigations. Extensive screening strategies aimed at detecting cancers in patients with unprovoked VTE, as per published studies, have not shown any significant improvement in patient survival rates [6].

The purpose of the present study was to investigate the in‐hospital mortality of patients with acute PE with either newly discovered cancer or previously known cancer, compared with patients with acute PE but without cancer. The association of newly or already known cancer‐associated PE to disease severity, bleeding, and the use of thrombolytic therapy was also investigated. We hypothesized that both groups of patients with cancer would have a higher in‐hospital mortality rate, more severe disease at presentation, a lower chance of being treated with thrombolytic therapy, and a higher incidence of major bleeding compared to patients without cancer.

## 2 Materials and Methods

We conducted a retrospective observational multicenter cohort study of the prospective registry of patients hospitalized due to acute PE verified with Computed Tomography Pulmonary Angiography (CTPA) imaging at admission to the hospital. The Regional Pulmonary Embolism Registry (REPER), consisting of eight hospitals (seven university hospitals and one general hospital), provided the data for the present study, which was conducted between January 2015 and December 2022. The diagnosis of acute PE was based on the European Society of Cardiology (ESC) algorithm, and all enrolled patients underwent CTPA [13, 14]. The majority of patients were evaluated in intensive care units upon admission. The registry did not include patients in the terminal stages of their chronic diseases.

- I patient group—patients with PE as the first manifestation of cancer. In 66 patients who were admitted to the hospital because of the newly presented signs and symptoms of PE, the diagnosis of cancer was established during the hospitalization for acute PE with imaging, endoscopy, and pathology assessments [15].
- II patient group—patients with PE and known cancer. 165 patients were treated for cancer within the previous 6 months (patients underwent curative or other types of surgery, received chemotherapy or immune therapy, had radiation procedures, or were treated with symptomatic therapy for cancer). All patients with known cancer had medical reports that confirmed the pathohistological diagnosis of cancer, localization, stage, TNM classification, and chemotherapy and/or radiotherapy protocol regimen applied.
- The control group—patients with PE without known cancer. 1514 patients were classified in this group with high probability in the following manner. There was no specific algorithm for the detection of cancer in this group of patients, and the approach to these diagnostic procedures was individualized with some general rules. For example, for patients with spontaneous PE, in addition to standard laboratory analysis and CTPA, blood tests for tumor markers, abdominal ultrasound, and CT of the chest with upper abdomen CT were performed. Additionally, women over 50 years of age were referred for gynecological and breast exams. Based on the medical history and laboratory tests that did not indicate cancer, as well as the results of the diagnostic procedures mentioned, it was concluded with a high probability that these patients did not have cancer and they were categorized into the group without known cancer. All patients were followed up within 3 months, and during that time, cancer did not occur in this group. Conversely, for patients in whom any of the above findings suggested cancer, further investigation was pursued and confirmed in certain cases. After that, those patients were classified into the I patient group. Patients with severe comorbidities, dementia, and elderly patients did not undergo previously mentioned additional diagnostic procedures to investigate the presence of a tumor.
- Exclusion subgroups. Thirty‐three patients who were in remission from cancer for more than 5 years, and 36 patients who had cancer within the last 5 years but had not undergone any specific cancer treatment for at least 6 months were excluded from the present study. This was first because these individuals have an increased risk of hidden cancer and require deep clinical and imaging workups, and second, the number of patients in this group was small and the addition of another complex group of patients would further complicate statistical analysis and decrease the reliability of the present study.

**FIGURE 1:**
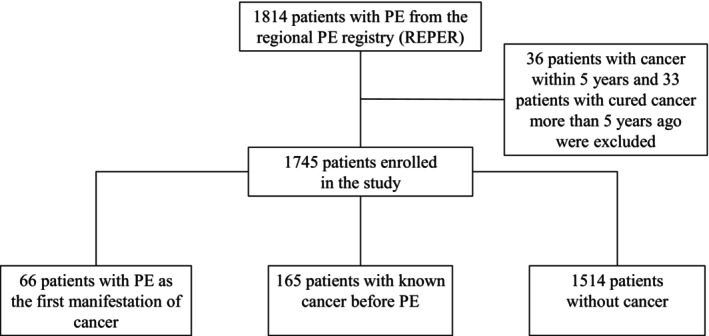
Flow chart of the study including patients with PE as the first manifestation of cancer, PE in patients with known cancer, and PE in patients without cancer as a comorbidity.

The flowchart of the study is presented in Figure 1.

### 2.1 Data Extraction

Trained physicians collected relevant data, including comorbidities, from the medical history of patients during their hospitalization. The oxygen saturation, systolic arterial pressure, heart rate, and basic laboratory blood levels of patients were measured upon admission to the hospital. A significant number of patients also underwent echocardiography imaging, measurement of cardiac troponin (cTn), and brain natriuretic peptide (BNP) blood levels on the first day of hospitalization (Table 1).

**TABLE 1:** Patient characteristics enrolled in the study. Laboratory and echocardiography parameters presented were obtained on the first hospitalization day.

Characteristics	PE as the first manifestation of cancer, *N* = 66	Known cancer, *N* = 165	Without cancer, *N* = 1514	*p*
Male sex, *N* (%)	19 (31.1%)	96 (43.2%)	188 (49.2%)	0.582
Age	64.58 ± 13.834	66.89 ± 11.186	63.17 ± 16.206	0.014
Age, *N* (%)				< 0.001
< 50 years	11 (16.7%) 55	12 (7.3%) 153	327 (21.6%)	
> 50 years	(83.3%)	(92.7%)	1187 (78.4%)	
Age, *N* (%)				0.689
< 75 years	52 (78.8%) 14	125 (75.8%)	1123 (74.4%)	
> 75 years	(21.2%)	40 (24.2%)	386 (25.6%)	
Comorbidities, *N* (%)				
CHF	10 (15.2%)	19 (11.5%)	227 (15%)	0.484
COPD	7 (10.6%)	22 (13.3%)	154 (10.2%)	0.453
Previous PTE/DVT	4 (6.2%)	9 (5.5%)	211 (14.1%)	0.002
Recent MB	5 (8.2%)	18 (8.1%)	29 (7.6%)	0.944
Smoking	13 (20.3%)	26 (16.7%)	262 (18.5%)	0.139
Diabetes mellitus	10 (15.4%)	32 (19.4%)	311 (20.5%)	0.575
Hypertension	36 (55.4%)	90 (54.9%)	920 (61.0%)	0.230
CAD	6 (9.2%)	19 (11.5%)	171 (11.3%)	0.866
Anemia (Hgb < 13 g/dL m. < 12 g/dL f.)	26 (39.4%)	83 (50.3%)	356 (23.5%)	< 0.001
Thrombocytopenia	1 (1.5%)	11 (6.8%)	54 (3.7%)	0.093
Recent surgery				< 0.001
0–21 day	4 (6.1%)	25 (15.2%)	189 (12.5%)	
21 days–6 months	5 (7.6%)	28 (17.0%)	70 (4.6%)	
Immobilization before PE^a^	9 (13.6%)	34 (20.6%)	276 (18.2%)	0.460
Severe chronic disability^b^	1 (1.5%)	10 (6.1%)	47 (3.1%)	0.095
Clinical characteristics				
HR on admission (bpm)	100.65 ± 21.19	102.53 ± 25.36	98.6928 ± 23.01	0.059
SBP on admission (mmHg)	120.91 ± 26.50	118.66 ± 22.58	123.68 ± 25.39	0.041
BMI (kg/m^2^)	25.01 ± 3.38	25.91 ± 4.20	27.69 ± 4.87	0.010
ESC mortality risk, *N* (%)				0.0001
Low	12 (18.2%)	29 (17.6%)	507 (33.5%)	
Intermediate–low	21 (31.8%)	54 (32.7%)	345 (22.8%)	
Intermediate–high	24 (36.4%)	52 (31.5%)	468 (30.9%)	
High	9 (13.6%)	30 (18.2%)	194 (12.8%)	
HAS‐BLED score	1.63 ± 1.12	1.57 ± 1.11	1.37 ± 1.137	0.031
Wells score	4.27 ± 2.38	4.55 ± 2.34	4.13 ± 2.43	0.104
Laboratory markers				
cTn × URL (ng/L)^c^	3.25 (0.33–12.73)	1.50 (0.44–11.57)	2.45 (0.48–9.90)	0.513
BNP × URL (pg/mL)^c^	3.51 (0.80–7.53)	2.21 (0.60–8.05)	2.11 (0.63–6.37)	0.374
S‐Creatinine (μmol/L)	94.38 ± 48.55	110.55 ± 76.85	105.32 ± 61.92	0.212
GFR (mL/min)	75.83 ± 32.79	68.95 ± 33.53	77.26 ± 36.88	0.033
D‐dimer (mg/L)	6.02 (3.11–13.95)	5.25 (2.50–10.00)	4.89 (2.50–9.53)	0.063
CRP (mg/L)	72.00 (37.30–131.10)	60.45 (29.45–132.00)	41.00 (15.8–89.80)	< 0.001
Glycemia (mmol/L)	6.40 (5.55–8.15)	6.70 (5.40–8.80)	6.80 (5.60–9.00)	0.383
PLT count 10^9^/L	241 (180–315)	213 (160–289)	221 (177–282)	0.171
Echocardiography^c^				
RV pressure (mmHg)	42.91 ± 19.72	45.22 ± 14.30	47.70 ± 18.19	0.062
RV basal diameter (cm)	3.42 ± 0.68	3.34 ± 0.84	3.47 ± 0.79	0.275
TAPSE (cm)	1.66 ± 0.33	1.84 ± 0.55	1.78 ± 0.47	0.279
EF‐left ventricle (%)	56.57 ± 11.25	59.55 ± 8.70	58.12 ± 10.15	0.139
Chemotherapy, *N* (%)		44 (26.7%)		
Radiation therapy, *N* (%)		14 (8.4%)		

Abbreviations: BMI, body mass index; BNP, brain natriuretic peptide; bpm, beats per minute; CAD, coronary artery disease; CHF, chronic heart failure; COPD, chronic obstructive pulmonary disease; CRP, C‐reactive protein; cTn, cardiac troponin; DVT, deep vein thrombosis; EF, ejection fraction; ESC, European Society of Cardiology; f., female; GFR, glomerular filtration rate; Hgb, hemoglobin; HR, heart rate; m., male; MB, major bleeding; PLT, platelet; PTE, pulmonary thromboembolism; RV, right ventricle; SBP, systolic blood pressure; TAPSE, tricuspid annular plane systolic excursion; URL, upper reference limit.

^a^ Immobilization—bed rest for more than 48 h, or because of traumatic injury.

^b^ Severe disability—hemiplegia, paraplegia, or long‐time bed‐ridden patients.

^c^ Missing values *N* (%) for PE as the first manifestation of Ca group, known cancer group and group without cancer: cTn‐33 (50.0), 75 (45.4), 547 (36.1), respectively; BNP‐37 (56.1), 80 (48.5), 638 (42.1), respectively; Echocardiography‐13 (19.7), 36 (21.8), 204 (13.5), respectively.

### 2.2 Risk Assessment

According to parameters at admission or during the hospitalization: hemodynamic state, CTPA finding (right ventricle (RV) to left ventricle (LV) diameter ratio > 1, and/or the estimation of thrombus burden), echocardiography assessment of RV function and diameters, and the elevation of blood levels of cTn and BNP, at the discretion of attending physicians, patients were finally categorized into four‐level stratums: high‐risk, intermediate‐high risk, intermediate‐low risk, or low‐risk patient groups, following the 2019 ESC PE guidelines [13, 14].

### 2.3 Echocardiographic Measurements

Echocardiographic imaging at presentation was encouraged to be performed in all patients where the examination was available. The echocardiographic exam was done as the initial imaging in patients with shock, and in the majority of patients, it was performed after the CTPA. The focus of the echocardiography was RV diameters and function, according to current guidelines [13].

### 2.4 Endpoints

Intra‐hospital death was selected as the primary endpoint, considering that both PE and cancer contribute to this outcome. The secondary endpoint of the present study was major and clinically relevant nonmajor bleeding during hospitalization, following the International Society of Thrombosis and Hemostasis definitions [16].

### 2.5 Statistical Analysis

All statistical analyses are performed using IBM Statistics SPSS version 20.0. The present study presented patient characteristics in terms of frequencies for categorical variables with either means ± standard deviation or medians with the 25–75 percentiles range, depending on the normality of the numerical variables. To test the differences between the three groups, the chi‐squared test, one‐way ANOVA with Bonferroni corrections for *p*‐values for multiple comparisons, or independent samples Kruskal–Wallis test with Bonferroni corrections for post hoc analysis were used, based on the normality distribution of variables. For the comparison of survival rates between three groups of patients, Kaplan–Meier curve analysis with a log‐rank test was employed. *p* < 0.05 was considered to indicate a statistically significant difference. A Cox regression analysis was performed to build a model for predicting in‐hospital mortality using the ESC risk model (low risk, intermediate‐low risk, intermediate‐high risk and high risk) together with the Charlson Comorbidity Index (CCI) in four grades (0, 1–2, 3–4, and > 4 points) [17, 18]. Points for malignant disease are not included in the CCI score because we tested our categorical variables in three groups (no cancer, known cancer, and newly diagnosed cancer). HR with 95% CI and *p*‐value of this adjusted multivariable model is presented. Binary regression analysis was used to determine the prognostic value of HAS‐BLED score > 1 for the prediction of the composite of major and clinically relevant nonmajor hospital bleeding.

## 3 Results

Out of a total of 1814 successive patients in the REPER, 1745 were enrolled in the present study based on the dependable history of cancer. Among them, 66 (3.7%) patients had PE as the first manifestation of cancer (group I), 165 (9.5%) patients had known cancer before PE (group II), and 1514 (86.8%) patients had no known cancer (group III). A flow chart of the present study is provided in Figure 1.

The relevant characteristics of patients across three predefined groups are presented in Table 1. There was no significant difference in the distribution between sexes across the three groups. Patients in the known cancer group were older than patients in the other two groups (*p* = 0.014). The distribution of patients younger than 50 years of age was significantly different between groups (*p* < 0.001). The group of patients with known cancer had the lowest number of younger patients. Furthermore, there were no significant differences in terms of the medical history of coronary artery disease, diabetes, arterial hypertension, chronic obstructive pulmonary disease, and chronic heart failure across the three groups. Also, the three groups did not differ in the frequency of immobilization before PE and severe chronic disability.

Patients without cancer had the highest BMI (*p* = 0.010), but admission glycaemia, serum creatinine, cTn, and BNP levels were similar between groups. Patients with known cancer had a lower glomerular filtration rate (GFR) than the other two groups (*p* = 0.033). C‐reactive protein was significantly higher in both cancer groups compared with the group without cancer. The basic echocardiographic parameters at admission were comparable between the three groups.

Data regarding the initial anticoagulant therapy and therapy on discharge are presented in Table S1.

There was a significantly different distribution of patients concerning ESC mortality risk stratification. Patients with known cancer had a higher frequency of high‐risk PE compared with patients without cancer (18.2% vs. 12.8%; *p* < 0.001). Patients with PE as the first manifestation of cancer had a higher frequency of intermediate‐high‐risk PE compared with the group of patients without cancer (36.4% vs. 30.9%; *p* < 0.001; Table 1).

The localization of cancer was different between the group with PE as the first manifestation of cancer and the group of patients with already diagnosed cancer. In the group of patients with PE as the first manifestation of cancer, pulmonary cancers predominated (37.9%), followed by urogenital (15.2%), gastrointestinal (15.2%), and carcinoma of unknown primary origin (7.6%). However, in the group of patients with known cancer, urogenital tumors dominated (26.1%), followed by gastrointestinal tumors (23.0%), breast cancer (17.0%) and pulmonary cancers (9.1%; Table 2). Among patients with known cancer, 44 (26.7%) were treated with anticancer drugs and 14 (8.4%) had undergone radiotherapy (in the last month).

**TABLE 2:** Localization of cancer in both cancer groups.

Localization of cancer	PE as the first manifestation of cancer, *N* (%) Σ*N* = 66	Known cancer, *N* (%) Σ*N* = 165
Pulmonary	25 (37.9%)	15 (9.1%)
Gastrointestinal	10 (15.2%)	38 (23.0%)
Colorectal	4	20
Stomach	1	7
Esophageal		2
Hepatic	1	5
Pancreatic	3	2
Other		2
Hematological	3 (4.5%)	13 (7.9%)
Urogenital	10 (15.2%)	43 (26.1%)
Renal‐bladder	1	15
Prostatic	3	9
Gynecological	6	19
Breast	2 (3.0%)	28 (17.0%)
Brain		8 (4.8%)
Other	2 (3.0%)	14 (8.5%)
Carcinoma of unknown primary	5 (7.6%)	3 (1.8%)
Not known	9 (13.6%)	3 (1.8%)
Metastatic	7 (10.6%)	39 (23.6%)

Patients with known cancer had a significantly lower chance of being treated with thrombolysis than patients without cancer (OR, 0.523; 95% CI, 0.339–0.807; *p* = 0.003); additionally, this difference remained when the OR was adjusted for age (OR, 0.542; 95% CI, 0.351–0.838; *p* = 0.006).

**FIGURE 2:**
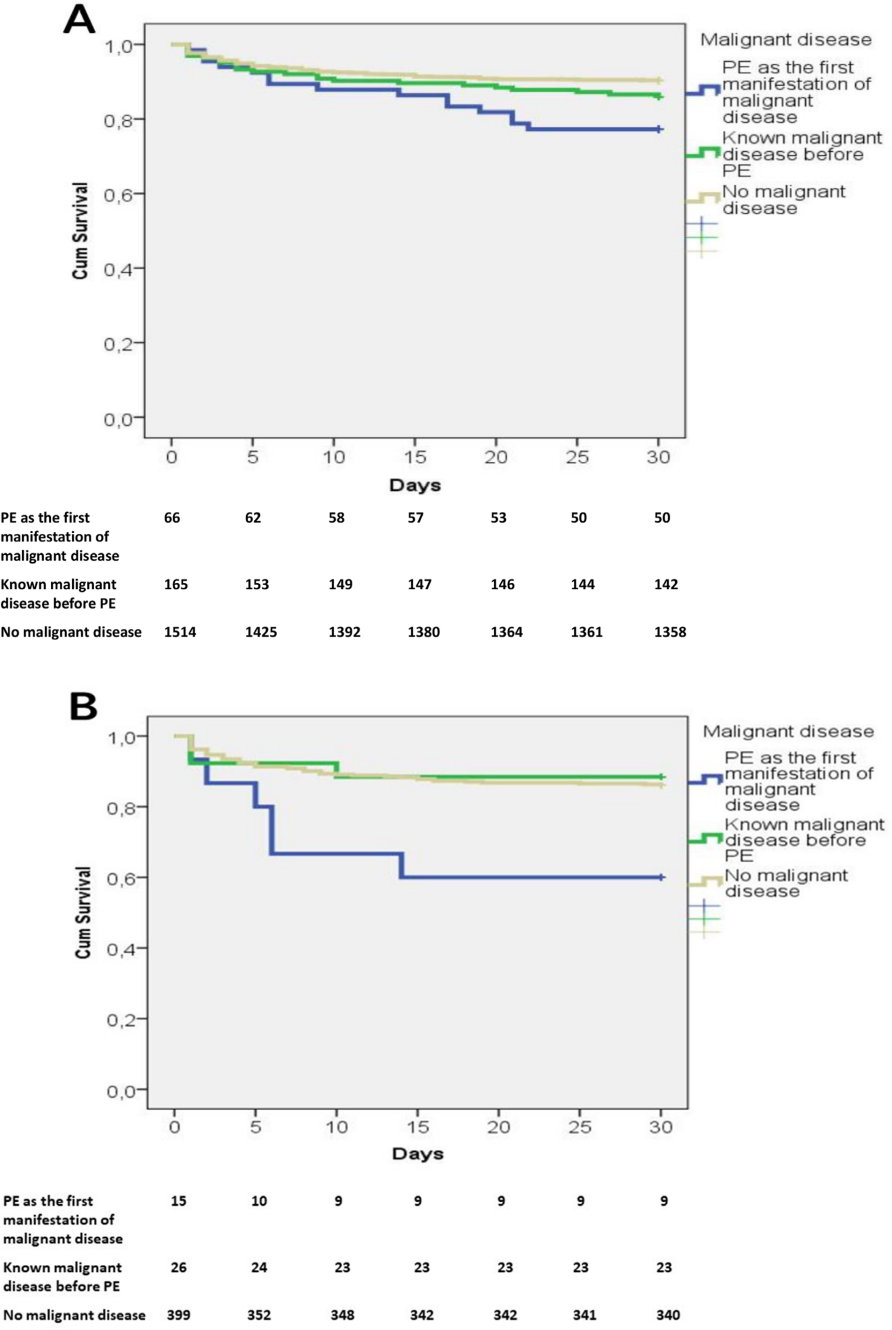
(A) Kaplan–Meier curves of survival across the three groups of patients. Patients with PE as the first manifestation of cancer had significantly higher hospital mortality rates than the group of patients with known cancer before PE and the group of patients without cancer (log‐rank *p* = 0.001). (B) Kaplan–Meier curves of survival across the three groups of patients who were treated with thrombolytic therapy. In patients who were treated with thrombolysis, patients with PE as the first manifestation of cancer had significantly higher hospital mortality than patients in the other two groups (log‐rank *p* = 0.011).

Kaplan–Meier analysis of hospital survival across the three groups is presented in Figure 2 (A‐all patients and B‐patients treated with thrombolytic therapy). Patients who had PE as the first manifestation of cancer had significantly lower hospital survival rates compared with the group of patients with known cancer and patients without cancer (77.3% vs. 86.0% vs. 90.3%; log‐rank *p* = 0.001). In patients who were treated with thrombolysis, patients with PE as the first manifestation of cancer also had a significantly worse prognosis than patients in the other two groups (hospital survival rate, 64.3% vs. 88.5% vs. 89.5%; log‐rank *p* = 0.011).

The duration from hospital admission to death was significantly longer in patients with PE as the first manifestation of cancer, with a median of 11 days (3–19 percentiles), compared to the group of known cancer before PE, with 5 days (2–18 percentiles), and those with no known cancer, with 4 days (2–10 percentiles); *p* = 0.037.

Patients with PE as the first presentation of cancer had the highest hospital mortality rate compared to the other two groups (HR for the mortality rate in patients without cancer as a reference, adjusted to four‐stratum ESC mortality risk and Charlson's comorbidity index (CCI) was 3.440; 95% confidence interval (CI), 1.795–6.591; *p* < 0.001). The univariate and multivariate Cox regression analyses are presented in Table 3, where it can be seen that PE as the first manifestation of cancer is an independent predictor of in‐hospital mortality, compared to ESC mortality risk and the CCI.

**TABLE 3:** Univariable and multivariable Cox‐regression model for the prediction of hospital mortality regarding the PE mortality risk, Charlson comorbidity index, and the status of malignant disease.

Variables	Univariable HR, 95% CI, *p*	Multivariable HR, 95% CI, *p*
ESC mortality risk		
Low‐risk	Reference	Reference
Intermediate–low risk	2.155, 1.109–4.187, 0.024	1.855, 0.952–3.614, 0.069
Intermediate–high risk	5.921, 3.352–10.458, < 0.001	4.871, 2.749–8.634, < 0.001
High risk	14.219, 8.009–25.246, < 0.001	11.801, 6.619–21.039, < 0.001
CCI in four grades^a^		
0 points	Reference	Reference
1–2 points	1.465, 0.691–3.104, 0.319	1.129, 0.532–2.398, 0.752
3–4 points	2.339, 1.153–4.747, 0.019	1.782, 0.885–3.629, 0.111
4 points	5.432, 2.743–10.757, < 0.001	3.757, 1.890–7.468, < 0.001
Status of malignant disease		
No cancer	Reference	Reference
Known cancer	1.460, 0.940–2.266, 0.092	1.186, 0.762–1.847, 0.450
PE as the first manifestation of cancer	2.580, 1.540–4.323, < 0.001	2.462, 1.467–4.130, 0.001

^a^ CCI was calculated without including points for malignant disease, as the status of malignant disease was used as a separate variable to assess its role in predicting hospital mortality in PE patients.

In the group where PE was the first manifestation of cancer, the cause of death was PE with acute cardiorespiratory failure associated with massive PE in 43.7% of patients, progression of cancer with multiorgan dysfunction (MODS) without hemodynamic compromise in 31.2%, and for the remaining patients, the causes were pneumonia with respiratory failure and sepsis (Table S2, along with the causes of in‐hospital mortality for the other two groups). Patients with known cancer had a higher comorbidity burden according to CCI compared to the other two groups (Table S3, *p* = 0.004). Patients with PE with known cancer had a higher hospital mortality rate compared to the group without cancer, but this difference did not reach a statistical difference (*p* = 0.08). The hospital mortality rate in the subgroup of patients who were treated with anticancer drugs was 15.9% and it is higher than in patients without cancer but similar to the rest of the group of patients with known cancer who were not treated with anticancer drugs.

In our study, the group of patients with PE as the first manifestation of cancer and the group of patients with known cancer had significantly higher HAS‐BLED scores compared to the no‐cancer group, as presented in Table 1. A HAS‐BLED score of 2 and higher had a prediction value for hospital composite of major and clinically relevant non‐major bleeding only in the group of patients without cancer (OR, 1.65, 95% CI, 1.142–2.402, *p* = 0.008). There was no significant difference in the composite of major and clinically relevant nonmajor hospital bleeding between groups (Table 4).

**TABLE 4:** Reperfusion treatment and hospital outcome of patients.

	PE the first manifestation of Ca, *N* = 66 (%)	Known Ca, *N* = 165 (%)	Without Ca, *N* = 1514 (%)	*p*	OR^1^	Adjusted OR^2^
Thrombolysis	15 (22.7)	26 (15.8)	399 (26.4)	0.011	0.822 (0.457–1.478)^3^ 0.523 (0.339–0.807)^4^	1.267 (0.558–2.878)^3^ 0.542 (0.351–0.838)^4^
*CDT* ^*5*^	*5* (*7.6*)	*7* (*4.2*)	*88* (*5.8*)			
ST^6^	1 (1.5)	0	2 (0.1)			
Hospital death	15 (22.7)	23 (13.9)	146 (9.6)	0.001	2.756 (1.512–5.024)^3^ 1.518 (0.946–2.434)^4^	2.749 (1.498–5.046)^3^ 1.427 (0.887–2.295)^4^
Hospital bleeding	7 (10.9)	7 (4.3)	123 (8.1)	0.146	1.385 (0.618–3.102)^3^ 0.506 (0.232–1.103)^4^	1.369 (0.611–3.068)^3^ 0.497 (0.228–1.083)^4^

*Note:* Odds ratio^1^, Odds ratio adjusted to age^2^. Odds ratio was presented as OR—comparison of PE as the first manifestation of carcinoma group^3^, and known carcinoma group^4^ to the group of patients without carcinoma. Catheter‐directed therapy^5^. Surgical thrombectomy^6^. Due to the small number of patients in these groups, statistical analysis was not performed for them. Italics values are subset of thrombolysis.

Abbreviations: Ca, cancer; PE, pulmonary embolism.

## 4 Discussion

There were three main results drawn from the present study. First, patients with PE as the initial manifestation of cancer had a much higher hospital mortality rate than patients without cancer; second, patients in both groups with cancer had more severe PE at presentation than patients without cancer; and third, patients with known cancer had less chance of being treated with thrombolysis than patients who did not have cancer or those who had not yet been diagnosed as having cancer.

According to the results of the present study, both groups of patients with cancer had higher hospital mortality rates, although that reached statistical significance only in the group with PE as the first manifestation of cancer. To explain the higher mortality rate among patients with cancer compared with patients without cancer, we provide the table “Causes of intrahospital mortality” as the Table S2. From these data, we can see that there is a weak trend toward higher PE‐related mortality (*p* = 0.183) in the group without cancer than in patients with cancer. Apart from PE, the most common underlying causes of death in both groups of patients with cancer were the progression of cancer and infections. The main causes of death in the group of patients with PE as the first manifestation of cancer were hemodynamic deterioration due to acute PE, multiorgan failure due to fulminant progression of cancer, and infections. These data underlie the possibility that many patients with newly diagnosed cancer after acute PE had very advanced cancer, which has a very poor prognosis. Acute PE is less often the main cause of death in this group of patients, and in concordance with that, the duration from admission to the hospital to death was significantly longer in this group of patients compared to the other two groups. In addition, it must be noted that according to the present study, both groups with cancer had more severe PE at presentation compared with patients without cancer. Although this result seems to be logical, patients with known cancer are often under careful surveillance for their cancer stage with serious various imaging, which might lead to the opposite—diagnosis of PE incidentally or at an earlier stage with a less severe presentation, like in the study of Himeno et al. who enrolled both symptomatic and incidentally discovered PE in cancer patients [19]. However, in our study, the inclusion criterion is symptomatic PE, patients with proven PE at CTPA, and hospitalization. Patients in the group with known cancer were older, had significantly lower GFR, and were more frequently anemic, especially in comparison with patients without cancer. In our study, 26.7% of patients with known cancer before PE were treated with anticancer therapy in the month preceding acute PE, and many of them were treated just a few days before acute PE manifested. The association between PE and anticancer therapy is possible. The hospital mortality rate in this subgroup of patients was 15.9%, which is higher than in the whole group but did not reach statistical significance, with the possible explanation that there were too small a number of patients in this subgroup.

On the other side, patients with PE as the first manifestation of cancer had the most pronounced inflammatory response with the highest CRP serum levels which might be a result of the combination of the more severe PE at presentation and advanced tumor nature. This marker is a strong predictor of RV dysfunction and higher hospital mortality rate in patients with acute PE [20, 21].

The majority of patients with newly diagnosed cancer had pulmonary primary or metastatic disease. This was an expected finding, as CTPA, which was used for the diagnosis of acute PE, can also result in the diagnosis of pulmonary tumors (imaging bias), and in the present study, the diagnosis of cancer in some cases was made almost inadvertently. On the other side, in some patients, we diagnosed very aggressive advanced lung cancers with also systemic influence, and in some patients, this combination of acute PE, advanced lung cancer, and systemic disturbances has some synergistic effect on the worse outcome. In these cases, pulmonary tumors might contribute to the diminished pulmonary reserve, and the two illnesses act synergistically. The advanced nature of newly diagnosed cancer disease in association with more severe acute PE at presentation, as a consequence of the high prothrombotic potential of these cancers, was possibly the main reason for the worst outcome in this group of patients. CTPA, abdominal ultrasound, and laboratory parameters indicative of cancer led to further testing and confirmed the diagnosis of advanced cancer in the newly diagnosed cancer.

In the group of patients with PE as the first manifestation of cancer, cancer was found during hospitalization because of symptoms caused by acute PE. This was opposite to the group of patients with known cancer in whom asymptomatic PE is diagnosed often using thoracic CT for tumor staging. It is also known that VTE in patients with cancer leads to a worse prognosis, probably due to the more malignant potential of such cancers, with some patients having unstoppable thrombotic processes that contribute to rapid death outcomes. The results of the present study in terms of the survival rate of patients with PE with known cancer were consistent with the literature. Our findings align with prior registry data from the COPE and GARFIELD‐VTE studies, which also emphasize the heightened risk of mortality in cancer‐associated PE [22, 23]. The COPE study demonstrated that patients with active cancer experience worse short‐term outcomes compared to those with previous cancer or no cancer, with a particularly high risk in those with metastatic disease [22]. Similarly, the GARFIELD‐VTE study confirmed that patients with active cancer are at greater risk of mortality, recurrent VTE, and bleeding, with VTE being the second leading cause of death [23]. No studies have been found that observed patients with PE as the first manifestation of cancer as an individual group. According to a recent study conducted by Becattini et al. [22], patients with active cancer had higher rates of in‐hospital and 30‐day mortality as compared with patients without cancer or with previous cancer. Kabrhel et al. identified cancer as one of the factors that were associated with all‐cause mortality within 30 days after PE [24]. Alotaibi et al. found that patients with cancer‐associated PE have a higher risk of all‐cause mortality in both short‐term (1 year) and long‐term (5 years) survival compared with those with unprovoked PE, in all age groups (*p* < 0.001) [25]. Furthermore, it is not only the diagnosis of cancer that worsens the survival rate of patients with PE, as PE can also worsen the survival rate of patients with cancer. The incidence of all‐cause mortality in hospitalized patients with cancer with PE was found to be 90% higher compared with patients with cancer without PE in a study conducted by Shalaby et al. [26].

In the present study, the group of patients with already diagnosed cancer was less likely to be treated with thrombolytic therapy independent of age compared with the other two groups, although notably, that was the group with the worst clinical presentation of PE. There are two possible reasons for these discrepancies. The first is that patients with known cancer had a higher frequency of recent surgery (32.2% of patients vs. < 20% in the other two groups). The second is a higher risk for bleeding in this group of patients, which makes physicians more cautious about using thrombolytic therapy. Although the HAS‐BLED score was not designed for the prediction of bleeding in acute PE where the use of parenteral anticoagulation therapy predominates, in our study, patients in both cancer groups had significantly higher HAS‐BLED scores compared to the no‐cancer group, which means that components of this score possibly influenced the decision of the use of systemic thrombolytic therapy. However, there are a lot of possible contributing factors for bleeding in patients with cancer beyond the components of the HAS‐BLED score, such as tissue damage by cancer or radio‐chemotherapy, thrombocytopenia, another hemorrhagic diathesis, various medical interventions, older age, anemia, etc. These factors represent strong markers for bleeding risk, which were much more frequently presented in the group of patients with known cancer. There is a limited amount of information available regarding the advantages and risks of thrombolytic treatment for patients with cancer with thrombosis [27]. In a recently published study, the usage of thrombolysis was lower in patients with PE with cancer in comparison with patients free of cancer regardless of clinical presentation [27]. A total of two groups of authors, using the same national inpatient database, reported results on lesser thrombolysis usage in the cancer population compared with those without cancer, which was most expressed in patients with metastatic disease [26, 28]. In a study by Weeda et al. [28], when the population was restricted to PE patients receiving thrombolysis at admission, no difference was observed in in‐hospital mortality between patients with cancer versus those without cancer.

In the present study, it was also found that patients with acute PE and newly diagnosed cancer who were treated with thrombolysis had the highest mortality rate, but the limited number of patients in this subgroup precludes any meaningful discussion, thus warranting further investigation. Advanced cancer and the high prothrombotic potential of these cancers might result in a worse prognosis of classic thrombolytic treatment in this subgroup. Nevertheless, a more cautious approach to reperfusion therapy, and perhaps the use of catheter‐directed therapy, might improve the efficacy and safety of reperfusion treatment in severe PE patients with cancer.

In the present study, the incidence of major bleeding during hospitalization was the lowest in patients with known cancer, and this was mainly because these patients were treated with thrombolysis significantly less than the other two groups. A recent study by Becattini et al. [22] reported a higher proportion of major bleeding in patients with active cancer and PE, regardless of used therapy, in comparison with those with previous cancer or without cancer (4.8%, 2.6%, and 2.4%, respectively). Furthermore, two groups of authors reported debatable results for the major bleeding rate in patients with cancer during thrombolytic therapy, including a 110% increased risk for major bleeding in the study by Jara‐Palomares et al. [29] and a nonsignificant risk in the study by Iskandar et al. [30]. This also implies that careful selection of patients with cancer who should be treated with thrombolysis and with what protocol can lower the risk of major bleeding in this vulnerable group of patients.

It is challenging to estimate the bleeding risk in patients with cancer receiving thrombolysis due to the lower use of fibrinolytic therapy in this group of patients [6]. Therefore, an individual patient‐based approach for the assessment of bleeding risk before thrombolysis treatment in patients with cancer is mandatory and recommended [27]. None of the 15 risk assessment models currently in use to evaluate the bleeding risk of patients with cancer proved to be validated in a recently released systematic review [31]. The CAT‐BLEED risk assessment approach is promising, but it has not yet undergone external validation and was created especially for patients with cancer with VTE receiving anticoagulant treatment [32]. The recently published novel PEBSI (Pulmonary Embolism Bleeding Score Index) score that consists of five basic characteristics (previous bleeding, diabetes mellitus and recent surgery as major factors, and the use of drugs that can cause bleeding and anemia as minor factors) was created for the detection of patients possessing a low risk for major bleeding on thrombolytic therapy [33]. The PEBSI score may have significant power to discriminate patients with low risk for major bleeding on thrombolytic therapy from those with high risk. However, further studies are required to determine whether it can be used in the population of cancer patients.

The present study had several limitations. A relatively small number of patients, especially in the subgroup of patients with acute PE as the first manifestation of cancer, precludes more powerful statistical analysis regarding outcome, clinical presentation, and bleeding risk. The outcomes of this study were in‐hospital mortality, the proportion of patients treated with thrombolysis and who had severe disease, and the occurrence of hospital bleeding classified according to the International Society on Thrombosis and Hemostasis (ISTH) criteria in hospitalized patients with pulmonary embolism. Over 100 variables were analyzed that could potentially influence these outcomes, including the presence of cancer. The diagnosis of cancer depends partly on the organization and level of a healthcare system, where well‐organized screening programs might detect earlier cancers, especially lung, colon, and urogenital carcinomas. For that reason, in the present study, the group of patients with acute PE as the first manifestation of cancer could not be representative of other healthcare systems. In the registry used in the present study, there were no special recommendations for the diagnostic procedures for establishing the diagnosis of cancer in patients who had no history of such diseases. In that way, a certain bias toward the diagnosis of pulmonary cancers (since CTPA is the main method for the diagnosis of acute PE) and more advanced cancers (with pronounced laboratory deviations or larger pathological findings on the various imaging methods) might be presented in this investigation. The main consequence of these limitations might be a higher hospital mortality rate in this group of patients with acute PE with newly diagnosed cancer. Another limitation is the exclusion of patients with a history of malignancy who were not treated for malignancy in the past 6 months. Although there was a high likelihood that these patients had malignant disease, complex diagnostic procedures to confirm malignancy were not feasible in the setting of acute PE. Moreover, since data on the type of chemotherapy were not available, its potential impact on the course of PE could not be assessed. To minimize bias, patient enrollment was performed independently by trained physicians across various institutions, and data analysis was conducted centrally using a standardized database. This process ensured consistent data entry and minimized bias related to study site variability.

Different cancers and stages of cancers might have very different impacts on the outcome, clinical presentation, management, and bleeding risk of acute PE. For that type of investigation, large registries or meta‐analyses may improve knowledge in the field.

The use of thrombolytic therapy was very high in the present investigation since the majority of patients in the REPER were admitted to the intensive care units. The use of thrombolytic therapy was at the discretion of the treating physicians, many of whom used thrombolytic therapy for both ominous and overt cases of hemodynamic collapse.

## 5 Conclusion

The clinical significance of the present study was that in patients in which PE was the first clinically significant sign of cancer, there was a dismal prognosis and high hospital mortality. Furthermore, patients with known cancer had less chance of being treated with thrombolytic therapy because of various absolute and relative contraindications for bleeding. Notably, in the registry used in the present study, there was no increase in major bleeding due to the presence of cancer. Further studies and analysis of registries may be useful in improving outcomes for these patients.

## Author Contributions

**Sonja Salinger:** conceptualization (lead), data curation (lead), formal analysis (lead), investigation (lead), methodology (lead), supervision (lead), writing – original draft (lead), writing – review and editing (lead). **Aleksandra Kozic:** conceptualization (lead), data curation (lead), formal analysis (lead), investigation (lead), methodology (lead), supervision (lead), writing – original draft (lead), writing – review and editing (lead). **Boris Dzudovic:** data curation (equal), formal analysis (equal), investigation (equal), writing – review and editing (equal). **Bojana Subotic:** data curation (equal), investigation (equal). **Jovan Matijasevic:** data curation (equal), formal analysis (equal), investigation (equal), writing – review and editing (equal). **Marija Benic:** data curation (equal), investigation (equal). **Vladimir Miloradovic:** data curation (equal), investigation (equal). **Ema Jevtic:** data curation (equal), investigation (equal). **Tamara Kovacevic‐Preradovic:** data curation (equal), investigation (equal). **Ljiljana Kos:** data curation (equal), investigation (equal). **Nebojsa Bulatovic:** data curation (equal), investigation (equal). **Bjanka Bozovic:** data curation (equal), investigation (equal). **Irena Mitevska:** data curation (equal), investigation (equal). **Marijan Bosevski:** data curation (equal), investigation (equal). **Ana Kovacevic‐Kuzmanovic:** data curation (equal), investigation (equal). **Milos Svircev:** data curation (equal), investigation (equal). **Aleksandar Neskovic:** data curation (equal), formal analysis (equal), investigation (equal), writing – review and editing (equal). **Bojan Mitrovic:** data curation (equal), investigation (equal). **Srdjan Kafedzic:** data curation (equal), investigation (equal). **Slobodan Obradovic:** conceptualization (lead), data curation (lead), formal analysis (lead), investigation (lead), methodology (lead), supervision (lead), validation (lead), writing – original draft (lead), writing – review and editing (lead).

## Ethics Statement

The study was conducted following the Declaration of Helsinki and approved by the Ethics Committee of the Military Medical Academy Belgrade, Serbia, for the constitution of the Regional Pulmonary Embolism Registry (REPER) and the protocol of data management under it. The present study was classified as a scientific project with approval number VMA‐MF‐01‐22‐24. Local review boards made permissions for each institution for participation in REPER and the use of data. During hospitalization, patients were informed about registry inclusion without personal data utilization. Verbal consent was obtained for privacy and ethical adherence.

## Conflicts of Interest

The authors declare no conflicts of interest.

## Supporting information


**Table S1.** Anticoagulant therapy in all three groups of patients.

**Table S2.** Causes of intrahospital mortality in all three groups.

**Table S3.** Status of cancer in PE patients and Charlson Comorbidity Index (CCI) in four strata.

## Data Availability

The data generated in the present study may be requested from the corresponding author.
