# Supplementary material for: Outcome of Patients With Cancer‐Associated Pulmonary Embolism: Results From the Regional Pulmonary Embolism Registry
Source: Cancer Med. 2025 Apr 25;14(9):e70886. doi: 10.1002/cam4.70886 (PMC12022772; doi:10.1002/cam4.70886)
Supplement: Supplementary file 1 — Table S1. Anticoagulant therapy in all three groups of patients. [file CAM4-14-e70886-s001.docx]

Supplementary Table I. Anticoagulant therapy in all three groups of patients.

|  | PE as the first manifestation of malignant disease,  N (%)  ΣN = 66 | Known malignant disease before PE, N (%)  ΣN = 165 | Without malignant disease, N (%)  ΣN = 1514 |
| --- | --- | --- | --- |
| **Initial anticoagulant therapy during hospitalization** |  |  |  |
| UFH | 28 (42.4%) | 51 (30.9%) | 561 (37.1%) |
| LMWH  *Enoxaparin*  *Nadroparin* | 36 (54.6%)  *29*  *7* | 98 (59.4%)  *79*  *19* | 857 (56.6%)  *724*  *133* |
| Fondaparinux | 0 | 7 (4.3%) | 13 (0.9%) |
| NOAC  *Rivaroxaban*  *Apixaban*  *Dabigatran* | 1 (1.5%)  *1* | 3 (1.8%)  *2*  *1* | 33 (2.2%)  *27*  *4*  *2* |
| Warfarin | 0 | 3 (1.8%) | 31 (2.0%) |
| Surgical thrombectomy | 1 (1.5%) | 0 | 2 (0.1%) |
| Without therapy | 0 | 2 (1.2%) | 11 (0.7%) |
| No data | 0 | 1 (0.6%) | 6 (0.4%) |
| **Anticoagulant therapy on discharge** | N (%)  ΣN = 50 | N (%)  ΣN = 142 | N (%)  ΣN = 1368 |
| LMWH | 16 | 44 | 77 |
| NOAC  *Rivaroxaban*  *Apixaban*  *Dabigatran* | 21  *8*  *11*  2 | 53  *24*  *21*  *8* | 794  *398*  *240*  *156* |
| Warfarin | 9 | 37 | 461 |
| No data | 4 | 8 | 36 |

UFH - unfractionated heparin, LMWH - low-molecular-weight heparin, NOAC - novel oral anticoagulants.
