# Supplementary material for: Outcome of Patients With Cancer‐Associated Pulmonary Embolism: Results From the Regional Pulmonary Embolism Registry
Source: Cancer Med. 2025 Apr 25;14(9):e70886. doi: 10.1002/cam4.70886 (PMC12022772; doi:10.1002/cam4.70886)
Supplement: Supplementary file 2 — Table S2. Causes of intrahospital mortality in all three groups. [file CAM4-14-e70886-s003.docx]

Supplementary Table II. Causes of intrahospital mortality in all three groups.

| **Causes of intrahospital mortality** | PE as the first manifestation of cancer  N (%)  ΣN = 16 | Known cancer before PE,  N (%)  ΣN = 23 | Without cancer  N (%)  ΣN = 146 |
| --- | --- | --- | --- |
| PE (the main cause was very probably PE with acute cardiorespiratory failure associated with massive PE)^1^ | 7 (43.7%) | 11 (47.8%) | 92 (63.0%) |
| Progression of malignant disease with multiorgan dysfunction (MODS) and without hemodynamic compromise | 5 (31.2%) | 5 (21.7%) | 0 |
| Sepsis | 2 (12.5%) | 3 (13.1%) | 8 (5.5%) |
| Bleeding | 0 | 0 | 10 (6.8%) |
| Other cause  *Acute kidney injury*  *Disseminated intravascular coagulopathy*  *Pneumonia and RF*  *Stroke*  *Respiratory failure*  *Acute myocardial infarction*  *Malignant ventricular arrhythmia*  *Acute left-sided heart failure*  *West Nile meningitis*  *Ketoacidosis*  *Acute respiratory distress*  *Neuroleptic Malignant Syndrome*  *Multiorgan dysfunction No data* | 2 (12.5%)  2 | 4 (17.4%)  3  1 | 36 (24.7%)  7  7  4  3  2  2  2  2  1  1  1  1  3 |
| Days from the admission to death  Median (25-75 percentile)^2^ | 11.5 (3.5-18.5) | 5 (2-18) | 4 (2-9) |

PE – pulmonary embolism, MODS – multiple organ dysfunction syndrome, DIK - disseminated intravascular coagulation, RF - respiratory failure, CVI - cerebrovascular insult, ACS – acute coronary syndrome, VF – ventricular fibrillation, ARDS - acute respiratory distress syndrome, MODS - multiple organ dysfunction syndrome.

^1^p=0.183, ^2^p=0.037
