# Supplementary material for: Outcome of Patients With Cancer‐Associated Pulmonary Embolism: Results From the Regional Pulmonary Embolism Registry
Source: Cancer Med. 2025 Apr 25;14(9):e70886. doi: 10.1002/cam4.70886 (PMC12022772; doi:10.1002/cam4.70886)
Supplement: Supplementary file 3 — Table S3. Status of cancer in PE patients and Charlson Comorbidity Index (CCI) in four strata. [file CAM4-14-e70886-s002.docx]

Supplementary table 3. Status of cancer in PE patients and Charlson Comorbidity Index (CCI) in four strata.

| Groups of patients | CCI 0 | CCI 1-2 | CCI 3-4 | CCI > 4 | p |
| --- | --- | --- | --- | --- | --- |
| No cancer  Known cancer  PE as the 1st manifestation of cancer | 209 (13.9%)  6 (3.6%)  8 (12.1%) | 412 (27.3%)  46 (27.9%)  20 (30.3%) | 471 (31.2%)  70 (42.4%)  23 (34.8%) | 417 (27.6%)  43 (26.1%)  15 (22.7%) | 0.004 |

The points of cancer are removed, because only the cancer comorbidities were aimed to test for the prognosis of hospital acute PE outcome.
